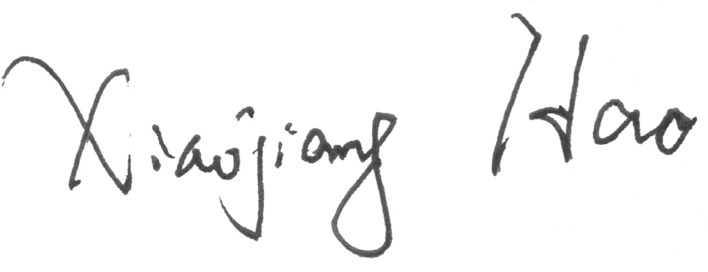# In Memory of the Late Professor Jun Zhou (1932–2020)

**DOI:** 10.1007/s13659-020-00296-4

**Published:** 2021-02-11

**Authors:** Xiao-Jiang Hao

**Affiliations:** State Key Laboratory of Phytochemistry and Plant Resources in West China, Kunming Institute of Botany, Chinese Academy of Sciences, Kunming, 650201 People’s Republic of China

It is my distinct privilege as Guest Editor of this special issue of *Natural Products and Bioprospecting* to honor the late Professor Jun Zhou with a diverse collection of Reviews, Original Research Articles and Short Communications on various aspects of plant natural products, wholeheartedly contributed by many of his former students, postdoctoral fellows, collaborators, colleagues and friends. These contributions serve as a reminder of the remarkable impact that an extraordinarily passionate individual can have on any chosen discipline.


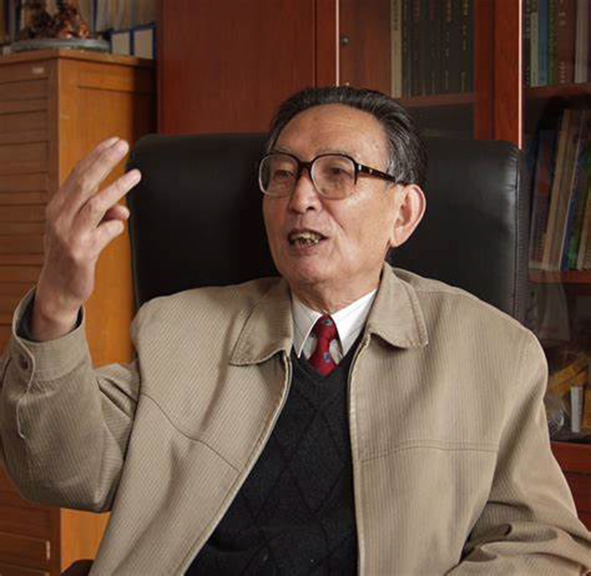


Zhou was born in 1932 in Dongtai County, Jiangsu Province, he attended a home school established by his eldest brother in 1945, the affiliated middle school of Jiang Danyang National Institute of Social Education in November 1946, the affiliated vocational school of Advanced Pharmacy of National Pharmaceutical College (predecessor of China Pharmaceutical University) in 1948, and studied Pharmaceutical Engineering at East China College of Chemical Technology (predecessor of East China University of Science and Technology) from 1954 to 1958 [1].

Zhou was a pioneer in studies of the phytochemistry and chemistry of traditional Chinese medicine in southwest China [1]. In September 1958, he joined the Plant Resource Chemistry Group of the Kunming Workstation of the Institute of Botany (predecessor of Kunming Institute of Botany established in April 1959), Chinese Academy of Sciences (CAS). Being equipped with a pickax and sickle, his first work experience involved a high yield test of *Pelargonium graveolens* L'Herit. He was involved in a survey of economic plants of southern and southeast Guizhou under instructions about the use and collection of China's wild plant raw materials issued by the State Council in 1958. He identified more than ten economic plants including *Blumea balsamifera* DC. (local producer of borneol), *Gaultheria fragrantissima* Wall. (its essential oil, i.e. wintergreen oil, mainly contains methyl salicylate) and *Cardiocrinum cathayanum* (E.H. Wilson) Stearn (Later he learned that its fruits are used as an alternative to *Aristolochia debilis* Siebold et Zucc. in Yunnan Province). This was my mentor’s beginning at learning botany and led him to become a half-botanist, a joking salutation by his colleagues afterwards. In the summer of 1960, CAS leaders assigned the project of food substitutes. He and colleagues investigated acorn nuts, *Chlorella* spp. and plant leaf proteins in Dali, Yunnan. They published ‘The distribution rules of tannins of the family Fagaceae in China’ in 1961 and the book entitled ‘Acorns’ [1]. During the cultural revolution (1966–1976), Prof. Zhou and co-workers investigated the origin of monocotyledons on the basis of chemical component comparison, reported triterpenoids from *Panax* L. and their relationship with the  taxonomy and geographical distribution [2], and isolated the new phenol, glucoside gastrodin, from *Gastrodia elata* Blume. Recently, it was reported that gastrodin could increase human reasoning abilities, lengthen sleep and decrease anxiety (https://mybiohack.com/blog/gastrodia-elata-blume-tianma-epilepsy-gaba). Overall, from 1958 to 2019, the Zhou laboratory took the lead in exploring plant glycosides and cyclic peptides in the genera *Panax*, *Paris*, *Gastodia*, *Cynanchum*, *Dioscorea*, *Acconitum* and the family Caryophyllaceae [1].

Zhou was the founder of the Department of Phytochemistry, the predecessor of the State Key Laboratory of Phytochemistry and Plant Resources in West China, Kunming Institute of Botany, CAS. This department was developed from the Plant Resource Chemistry Group that was established in 1958, and which he joined. Soon after his arrival, he was named secretary of this group by Prof. Xi-Tao Cai, Director of the Kunming Institute of Botany. In 1964, the Department of Phytochemistry was founded. From then on, Zhou was the virtual academic leader of the department. Indeed, he was the executive leader of the department from 1964 to 1986.

Zhou was a mentor in the Department of Phytochemistry, Kunming Institute of Botany, CAS. He recognized that the development of science depends on cultivating talents. He obtained funding from several Japanese pharmaceutical companies to support the training of more than ten young colleagues as visiting scholars and/or PhD students at the University of Tokushima, Hiroshima University, Hokkaido University and Kyoto University. He impartially helped his co-workers to establish their own research fields. Thus, the Kunming Institute of Botany is well known for phytochemistry, particularly in the fields of steroidal saponins, triterpene saponins, triterpenoids [1], diterpenoids, and diterpene alkaloids [3]. It shall be engraved that to Zhou’s great merit, he raised a generation of young researchers into fine scientists who contributed to making the State Key Laboratory of Phytochemistry and Plant Resources in West China a center of scientific excellence. They now mourn Jun Zhou—a scientist, mentor and friend.